# Machine learning prediction of adolescent HIV testing services in Ethiopia

**DOI:** 10.3389/fpubh.2024.1341279

**Published:** 2024-03-15

**Authors:** Melsew Setegn Alie, Yilkal Negesse

**Affiliations:** ^1^Department of Public Health, School of Public Health, College of Medicine and Health Science, Mizan-Tepi University, Mizan-Aman, Ethiopia; ^2^Department of Public Health, College of Medicine and Health Science, Debre-Markos University, Gojjam, Ethiopia

**Keywords:** HIV, machine learning, adolescent, Ethiopia, ML

## Abstract

**Background:**

Despite endeavors to achieve the Joint United Nations Programme on HIV/AIDS 95-95-95 fast track targets established in 2014 for HIV prevention, progress has fallen short. Hence, it is imperative to identify factors that can serve as predictors of an adolescent’s HIV status. This identification would enable the implementation of targeted screening interventions and the enhancement of healthcare services. Our primary objective was to identify these predictors to facilitate the improvement of HIV testing services for adolescents in Ethiopia.

**Methods:**

A study was conducted by utilizing eight different machine learning techniques to develop models using demographic and health data from 4,502 adolescent respondents. The dataset consisted of 31 variables and variable selection was done using different selection methods. To train and validate the models, the data was randomly split into 80% for training and validation, and 20% for testing. The algorithms were evaluated, and the one with the highest accuracy and mean f1 score was selected for further training using the most predictive variables.

**Results:**

The J48 decision tree algorithm has proven to be remarkably successful in accurately detecting HIV positivity, outperforming seven other algorithms with an impressive accuracy rate of 81.29% and a Receiver Operating Characteristic (ROC) curve of 86.3%. The algorithm owes its success to its remarkable capability to identify crucial predictor features, with the top five being age, knowledge of HIV testing locations, age at first sexual encounter, recent sexual activity, and exposure to family planning. Interestingly, the model’s performance witnessed a significant improvement when utilizing only twenty variables as opposed to including all variables.

**Conclusion:**

Our research findings indicate that the J48 decision tree algorithm, when combined with demographic and health-related data, is a highly effective tool for identifying potential predictors of HIV testing. This approach allows us to accurately predict which adolescents are at a high risk of infection, enabling the implementation of targeted screening strategies for early detection and intervention. To improve the testing status of adolescents in the country, we recommend considering demographic factors such as age, age at first sexual encounter, exposure to family planning, recent sexual activity, and other identified predictors.

## Introduction

In 2016, 2.1 million adolescents were infected with HIV, with 1.7 million in sub-Saharan Africa (1). Effective antiretroviral treatment has decreased perinatal HIV infection, but challenges remain in treatment and care. Sub-Saharan Africa has the highest burden of HIV, with young women at higher risk (2–4). HIV testing is crucial for diagnosis, treatment, and prevention, including the prevention of mother-to-child transmission. A well-functioning HIV testing service is essential for reducing HIV-related illnesses and deaths (5–7).

HIV testing is a crucial public health program aimed at reducing the spread of HIV/AIDS and mitigating its impact on communities and national economies (8, 9). It serves as a critical entry point for HIV detection, care, treatment, prevention, and support services (10, 11). HIV testing protects individuals who have been exposed to an HIV-positive partner and their infants from infection (12). The Sustainable Development Goals prioritize ending the HIV/AIDS epidemic by 2030, making prevention and control of the disease a critical agenda item (13). Studies show that HIV testing is the most cost-effective measure for preventing and controlling HIV transmission in Africa (14, 15). The Ethiopian government has embraced voluntary HIV counseling and testing as a key component of the country’s HIV/AIDS prevention and control efforts (16).

Regular HIV testing is crucial for meeting the Joint United Nations Programme on HIV/AIDS (UNAIDS) 95-95-95 targets by 2030, which aim to ensure that 95% of people living with HIV are diagnosed, 95% of those diagnosed are receiving antiretroviral therapy (ART), and 95% of those on ART are virally suppressed (17). In 2016, the World Health Organization (WHO) expanded ART eligibility guidelines to ensure that all people living with HIV have access to treatment, with a focus on same-day ART initiation whenever feasible. This approach, known as “test and treat,” is an essential step towards achieving universal ART coverage and ending the HIV epidemic (18).

Despite women being at a higher risk of HIV infection, access to HIV testing remains uneven, particularly in sub-Saharan Africa where the prevalence of HIV among adults is alarmingly high. The uptake of HIV testing and counseling among women, including adolescent girls, remains low, even though they account for two-thirds of new infections globally (19–23). In Ethiopia, HIV voluntary counseling and testing (VCT) has been a key strategy in the country’s efforts to prevent and control HIV/AIDS. However, the utilization of VCT services among males, females, adults, and rural residents in Ethiopia is still inadequate (24).

Adolescents face barriers in accessing information and services related to HIV and reproductive health due to factors like age and socioeconomic status (22, 25). Given the high HIV burden in many countries, adolescence presents an opportunity for early intervention. Comprehensive data is essential for shaping accurate HIV-related messages and services before risky behaviors become entrenched. Socioeconomic and structural challenges such as poverty, limited education, and gender inequality can increase the risk of HIV infection (26, 27). While risk awareness is important, it is not enough to address the challenges of HIV/AIDs (5). Having a positive attitude towards HIV knowledge is influenced by factors like education, social status, and gender, which can contribute to better sexual and reproductive health policies (6). Previous studies conducted in various regions of the world have identified several factors that are significantly associated with HIV testing among men. These factors include marital status, age, educational level, region of residence, having multiple sexual partners, wealth index, condom use, exposure to mass media, and age at first sexual encounter (25, 27–31). Additionally, stigma towards HIV patients, comprehensive HIV knowledge, and engaging in risky sexual behavior have been found to have positive associations with HIV testing and counseling (6, 19, 22, 28, 31, 32).

The global coverage of HIV testing and counseling among adolescents is currently low (16). In Africa, where the age of first sexual encounter is decreasing, many adolescents are also contracting other sexually transmitted infections (STIs) such as gonorrhea, chlamydia, and (33) syphilis, which can potentially increase the risk of acquiring and transmitting HIV (2). Additionally, the lack of knowledge among adolescents about STI symptoms and modes of transmission further compounds these health challenges (17). While there have been a few studies conducted on these issues in Ethiopia (7, 8), there is currently no study in the country that assesses HIV testing and counseling by using a machine learning algorithm. The use of algorithm-based prediction for HIV testing and identifying the most influential factors in adolescent HIV testing is a novel approach that has not been explored yet. Currently, there is a lack of research on machine learning prediction of adolescent HIV testing specifically in Ethiopia. Therefore, the main objective of this study is to utilize machine learning techniques to predict HIV testing and counseling among adolescents aged 15–24 years in Ethiopia.

## Methods

### Patient selection

The research-utilized data from a publicly available demographic and health survey conducted in the country. Out of the 12,688 data points collected, 8,186 were excluded from the study as they pertained to individuals who were either younger than 15 or older than 24 years old. Following the exclusion of these data points, the analysis concentrated on a group of 4,502 adolescents who were included in the study.

### Outcome variable

In this study, we referred to the outcome variable as “tested,” which indicated whether the adolescent had undergone HIV testing within the past 5 years. If the adolescent had been tested during the survey period, we coded the variable as “Yes.” On the other hand, if the adolescent had not been tested for HIV within the past 5 years, we coded the variable as “No”.

### Data preprocessing

In this specific study, we made the deliberate choice to include individuals aged 15–24 years old. We extracted from publically available data set of demographic and health survey of 2016.^1^ Then we filtered the data of adolescent in this data set. Our data was sourced from a de-identified demographic and health survey database, encompassing information from a total of 4,502 adolescent individuals. To ensure the integrity of our data, we enlisted the expertise of two epidemiologists who collaborated on the project. Their valuable insights were instrumental in identifying and addressing any discrepancies such as noisy or abnormal values, errors, duplicates, and irrelevant data. Additionally, we meticulously reviewed the initial list of parameters to guarantee consistency in the data preprocessing stage. Ultimately, our analysis focused solely on the data pertaining to the 4,502 adolescents within the age range of 15–24 years old. To enhance clarity and coherence, we assigned appropriate labels to both nominal and continuous variables based on previous literature.

### Random forest

The random forest (RF) algorithm is known for significantly improving the classification accuracy of a model. This is achieved by generating multiple decision trees. Each decision tree produces a result for a given sample, and the final result is determined based on the majority of the decision trees’ results (34). To maximize the performance of the RF algorithm, we conducted hyperparameter tuning and 10-fold cross-validation for all our experiments. We focused on tuning the same set of hyperparameters. These hyperparameters include the maximum depth values of the decision trees and the minimum number of samples required in a leaf node. In order to assess the quality of each node’s split, we used two criteria: the Gini index and entropy. Since the RF algorithm generates multiple decision trees, we utilized the “number of estimators” hyperparameter to control the number of trees created in the forest. The values we experimented with for this hyperparameter were 100, 200, and 300. It’s worth noting that each decision tree in the RF algorithm learns from random subsets of samples drawn from the dataset. The use of bootstrap sampling, which involves drawing samples with replacement, ensures the diversity and robustness of the decision trees. Additionally, we tuned the “bootstrap” hyperparameter, which determines whether the entire dataset is used to build a decision tree. When set to “False,” the bootstrap parameter ensures that the entire dataset is used. On the other hand, setting it to “True” allows bootstrap sampling to take place. By carefully tuning these hyperparameters, we aimed to achieve the highest possible performance for the RF algorithm in our experiments.

### Imbalanced data handing

In machine learning, dealing with imbalanced data can be a significant challenge. This occurs when the distribution of classes in a dataset is uneven, which can lead to biased results in favor of the majority class. In the current dataset being analyzed, there is a substantial imbalance between the “not tested” and “tested” classes, with 2,950 and 1,552 cases, respectively. This imbalance can lead to inaccurate results and make it likely for new observations to be categorized into the majority class. To address this issue, the study utilized a method called synthetic minority over-sampling technique (SMOTE) from the imbalanced-learn toolbox. SMOTE generates synthetic samples for the minority class by interpolating between existing minority class samples. By applying SMOTE, the dataset was balanced, allowing for more accurate and unbiased training of machine learning models. If you are interested in learning more about the imbalanced-learn toolbox and the SMOTE method, you can visit their website at https://imbalanced-learn.org/stable/.

Predictive accuracy is a commonly used metric to evaluate the performance of machine learning algorithms. However, when working with imbalanced datasets, accuracy can be misleading and hinder the identification of underlying causes such as HIV testing. In this particular study, researchers employed various techniques to address the class imbalance issue in their dataset. They utilized Synthetic Minority Oversampling Technique (SMOTE) (35), which generates new samples by interpolating between existing samples and their neighbors (36, 37). Additionally, they employed random under-sampling, which involves discarding samples from the majority class until the minority class reaches a predetermined percentage of the majority class (35). Another method used was Adaptive Synthetic (ADASYN), which generates synthetic data for harder-to-learn minority class samples, thereby reducing bias introduced by imbalanced data distribution. Through the application of these techniques, the researchers successfully achieved a balanced dataset (38). The outcome of their endeavors is discussed in detail in the result section of the study (39).

### Feature selection

In the initial phase of our study, our primary aim was to identify key features that could accurately predict the HIV testing status of adolescents in Ethiopia. To achieve this, we conducted a thorough review of scientific literature by searching various databases. The findings from this review were then used to create a comprehensive questionnaire, which covered a wide range of predictors for HIV testing services. By incorporating these identified predictors, we aimed to develop a reliable tool for predicting HIV testing status. The SHAP analysis was done for the features. SHAP is a powerful technique that is independent of any specific machine learning model. It is used to calculate the Shapley values of the different features in a model, providing explanations for the model’s predictions. SHAP feature importance is determined by taking the average of the absolute values of the Shapley values for each feature. This approach allows for a comprehensive understanding of the impact each feature has on the model’s predictions.

**Figure 1:**
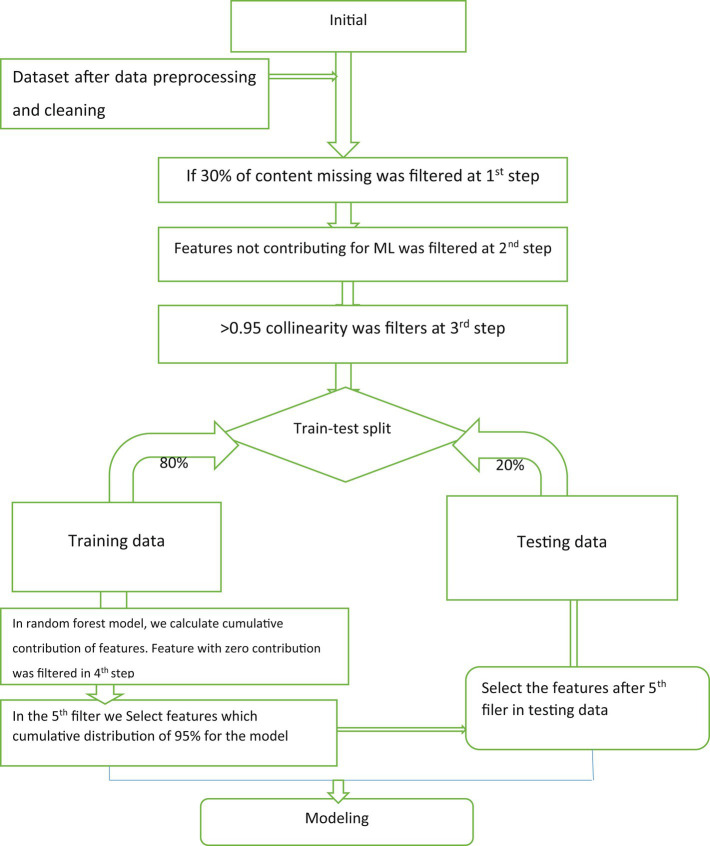
The flowchart of variable selection for machine-learning algorithm model.

During this stage, our main objective was to identify specific features that could accurately predict HIV testing status. We began by conducting a comprehensive review of scientific databases to determine the most relevant features. Based on this review, we developed a questionnaire that included predictors. We carefully compiled all the pertinent information to create the final version of our data collection tool. By doing so, we aimed to ensure that our study would yield accurate and reliable results that could help inform future efforts to improve HIV testing services in Ethiopia. WEKA version 3.9 was used to select important features, R version 4.0.2 and python version 3.2 was used for the analysis of data. Figure 1 displays a flowchart that outlines the process used to select the final variables for a machine-learning model. This process involved five distinct steps. The first step involved removing features from the dataset that had a missing value greater than 30%. In the second step, features that were deemed irrelevant to the final outcome variable, such as reference date, patient ID, and accompanying information, were eliminated. The third step addressed collinearity, which can lead to duplicated features and skew the model’s results. Features with a collinearity greater than 0.95 were removed from the dataset. By implementing these procedures, the most relevant and informative features for the machine learning model were identified, as shown in Figure 1. In this study, various feature selection methods were employed to identify the most relevant predictive features. These methods included recursive feature elimination (RFE), random forest feature importance, and the Boruta feature selection method. RFE is a technique used for feature selection, which begins with all the features in the training dataset and iteratively eliminates features until the desired number of features is reached. This method is particularly effective in reducing model complexity and improving the efficiency of machine learning algorithms. By utilizing these feature selection methods, the study aimed to identify the most informative features that contribute significantly to the predictive power of the model (40). This approach helps to streamline the data processing and enhance the accuracy of machine learning algorithms.

### Model development

A comprehensive literature review was conducted to develop accurate predictive classifier models for HIV testing status (19, 33, 41–45). The selection of suitable machine learning (ML) algorithms was based on the type and quality of the dataset utilized. Eight ML algorithms were employed to construct the individual testing prediction model: J48 decision tree, random forest (RF), k-nearest neighborhood (k-NN), Support Vector Machine (SVM), multi-layer perceptron (MLP), Naïve Bayes (NB), logistic gradient boosting (logit Boost), and logistic regression (LR). The data were analyzed using Weka software v3.9.2 python, and R software to implement the algorithms, analyze and calculate curves and criteria, and draw the confusion matrix.

### Cross-validation

**Figure 2:**
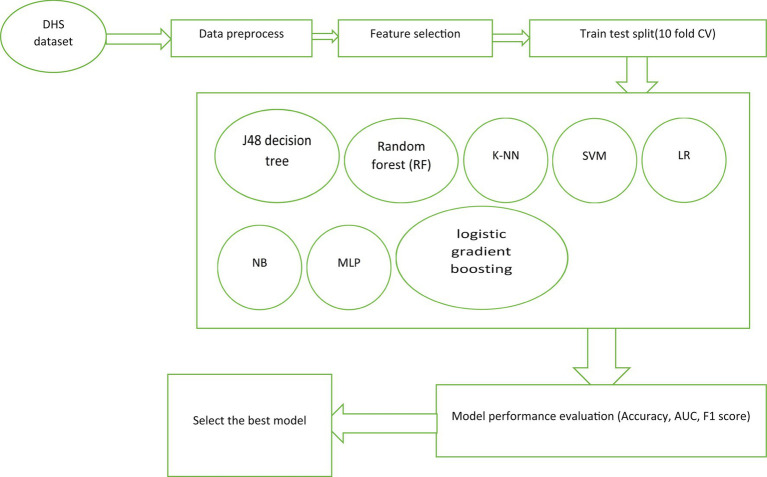
Workflow of machine learning for adolescent HIV testing prediction.

We used the EXPLORER module in WEKA to find the best hyperparameters for our models and evaluated their performance using tenfold cross-validation. We ran experiments with WEKA’s EXPERIMENTER module and repeated the cross-validation process to ensure reliable results. We used an 80:20 ratio for training and testing, and calculated average performance metrics across ten runs. We choose stratified tenfold cross-validation to accurately estimate accuracy. Our approach aims to minimize errors and biases by using more data for training and validation. We used WEKA’s EXPLORER and EXPERIMENTER modules along with tenfold cross-validation for a robust evaluation and comparison of classification models. We selected stratified tenfold cross-validation as it strikes a favorable balance between bias and variance, making it a preferred technique for accurately estimating accuracy. It is worth emphasizing that tenfold cross-validation is widely employed in the fields of machine learning and data mining due to its advantages over traditional instance splitting methods. This provides a reliable and robust method for evaluating and comparing the effectiveness of classification models. The summary of machine learning pipeline presented on Figure 2.

### Model evaluation

Evaluating the performance of a machine-learning model is essential for its success. In our study, we thoroughly assessed the performance of our predictive models using various performance metrics, as outlined in Table 1. These metrics encompassed accuracy, specificity, precision, sensitivity, and the receiver operating characteristic (ROC) chart criteria. By leveraging these metrics, we effectively measured the effectiveness of our models in predicting HIV testing services of adolescents. To determine the best model for predicting HIV testing service of adolescent, we compared the performance of each model using the aforementioned evaluation criteria. The results of this comparison are summarized in Table 2. Through a meticulous analysis and comparison of these evaluation criteria, we successfully identified the model that demonstrated the highest performance in predicting HIV testing. Our comprehensive evaluation process enabled us to select the most effective model and gain valuable insights and confidence in its predictive capabilities.

**Table 1:** Confusion matrix.

Output	Predicted value	
Actual value	Tested (+)	Not tested (−)
Tested (+)	True positive (TP)	False negative (FN)
Not tested (−)	False positive (FP)	True negative (TN)

**Table 2:** The performance evaluation measures.

Performance criteria	Calculation
Accuracy	(TP + TN)/(TP + TN + FP + FN)
Sensitivity/recall	TP/(TP + FP)
Precision	TP/(TP + FN)
Specificity	TN/(TN + FP)
F1 score	$\frac{2^{*}precision^{*}recall}{Precision+recall}$

### Association rule

The technique of association rule mining examines the correlations between multiple variables in a group, and it was first developed by Agarwal and Srikanth (46). In this study, an additional method was utilized to support the classification of machine learning algorithms for predicting adolescent HIV testing using R software. The apriori algorithm was employed to uncover associations between the selected features and the target feature (47). A minimal support degree of 0.00095 and a minimum confidence threshold of 90% were set to identify all potential association rules. This is because a rule is considered reliable if its confidence level is more than 80% (48). In this study, the main focus was on identifying features that are associated with adolescent HIV testing through the use of association rules. Specifically, the study utilized a technique known as classification association rules (49), which involves analyzing the features that are implied by the target features (Antecedent = > Consequent). The goal of this approach was to classify all the variables that contribute to HIV testing among adolescents and to identify the predictors that each category contributes to the testing. To evaluate the strength of each rule, the study used the metrics of Support, Confidence, and Lift. It is important to note that in this context, the sets of features represented by X and Y are mutually exclusive.

Rule X = > Y:

Support = $\frac{\mathrm{Fequence}\;\left(\mathrm{X,}\;\;Y\right)}{N}$ , Confidence = $\frac{\mathrm{Frequency}\;\left(\mathrm{X,Y}\right)}{\mathrm{Fequency}\;\left(X\right)}$ , Lift = $\frac{\mathrm{Frequency}\;\left(\mathrm{X,Y}\right)}{\mathrm{Fequency}\;\left(X\right)\;\mathrm{Frequency}\;\left(Y\right)}$

## Results

### Patient characteristics and descriptive statistics

After applying our exclusion criteria and conducting a quantitative analysis of case records, we have identified a total of 4,502 adolescents in the database who met the eligibility criteria for our study. Among these participants, 3,339 (74.2%) adolescents were male, while 1,163 (25.8%) adolescents were female. The mean age of the study participants was 19.09 (±2.841) years old. A majority of the study participants, 3,137 (69.7%), reported residing in rural areas. Additionally, 55.2% of the total 4,502 study participants had attended primary education. An overwhelming majority, 4,292 (95.3%) of the adolescents, had previously heard about AIDS. Out of the total study participants, 79.4% of them knew the place for HIV testing. Regarding awareness of sexually transmitted infections (STIs), the majority (95.7%) of the adolescents were aware of STIs (Table 3).

**Table 3:** Descriptive statistics of the current study conducted in Ethiopia.

Feature	Value	Frequencies	Feature	Value	Frequencies
Residence	Rural	3,137 (69.7%)	Age	Mean ± SD	19.09 ± 2.841
	Urban	1,365 (30.3%)			
Educational level	No education	513 (11.4%)	Total children ever born	Zero	4,207 (93.4%)
	Primary	2,484 (55.2%)			
				> = 1 child	295 (6.6%)
	Secondary	1,102 (24.5%)			
	Higher	403 (9.0%)			
Region	Tigray	504 (11.2%)	Number of household member	1–3 members	979 (21.7%)
	Afar	252 (5.6%)			
				4–6 members	1876 (41.7%)
	Amhara	588 (13.1%)			
	Oromia	603 (13.4%)		≥7 members	1,647 (36.6%)
	Somali	378 (8.4%)			
	Benishangul	327 (7.3%)			
	SNNPR	569 (12.6%)			
	Gambella	338 (7.5%)			
Sex of household head	Male	3,339 (74.2%)	Number of living children	Have no child	4,219 (93.7%)
	Female	1,163 (25.8%)		One and above	283 (6.3%)
Literacy	Cannot read at all	839 (18.9%)	Recent sexual activity	Never had sex	3,166 (70.3%)
	Able to read only part of sentences	729 (16.2%)			
				Active in the last 4 weeks	622 (13.8%)
	Able to read whole part of sentences	2,878 (63.9%)			
				Not active in the last 4 weeks	714 (15.9%)
	No card on required language	53 (1.2%)			
	Blind/visual impaired	3 (0.1%)			
Wealth index	Poorest	993 (22.1%)	Currently working	Yes	1,519 (33.7%)
	Poorer	608 (13.5%)			
	Middle	625 (13.9%)		No	2,983 (66.3%)
	Richer	759 (16.9%)			
	Richest	1,517 (33.7%)			
Knowledge of ovulatory cycle	Did not have knowledge	1,097 (24.4%)	Awareness on STI	Yes	4,307 (95.7%)
	Have knowledge	3,405 (75.6%)		No	195 (4.3)
Knowledge of any contraceptive method	Know no method	209 (4.6%)	Worked in last 12 months	No	1,257 (27.9%)
	Know any methods	4,293 (95.4%)		In past year	262 (5.8%)
				Currently working	2,983 (66.3%)
Respondent circumcised	No	455 (10.1%)	Ever heard of AIDS	Yes	4,292 (95.3%)
	Yes	4,033 (89.9%)			
	I do not know	14 (0.3%)		No	210 (4.7%)
Current marital status	Never in union	4,014 (89.2%)	Alcohol drinking	Yes	1764 (39.3%)
	Married	488 (10.8%)		No	2,722 (60.7%)
Age at first sex	Not had sex	3,166 (70.3%)	Intimate partner violence	Yes	1,472 (32.7%)
	Between 8 and 15 years	248 (5.5%)		No	3,030 (67.3%)
	Between 16 and 24 years	1,088 (24.2%)			
Ever been tested for HIV	Yes	1,552 (34.5%)	Resource control	Yes	901 (20.0%)
	No	2,950 (65.5%)		No	3,601 (80.0%)
Know a place to get HIV test	Yes	3,576 (79.4%)	Media exposure	Yes	3,108 (69.0%)
	No	926 (20.6%)		No	1,394 (31.0%)
Have you ever chewed chat?	Yes	3,648 (81.0%)	Fear of Stigma and discrimination	Yes	4,046 (94.3%)
	No	854 (19.0%)		No	246 (5.7%)
Exposure to family planning	Yes	2,768 (61.5%)	Number of Sexual partner	Have no sexual partner	2,924 (87.2%)
				Only one	487 (10.8%)
	No	1734 (38.5%)		Two and above	91 (2.0%)
Smoking status	Yes	71 (1.6%)	Health insurance	Yes	4,295 (95.4%)
	No	4,431 (98.4%)		No	207 (4.6%)

### Data pre-processing results

In this particular study, various pre-processing steps were undertaken to handle missing or null values, encode categorical labels, and balance the dataset. Initially, the incomplete raw data and missing values were addressed using the imputation technique. Supplementary Figure S1 provides a breakdown of the percentage of missing values for each feature, which shows that the feature know the place of HIV testing 210 (4.66%), alcohol drinking 16 (0.3554%), and stigma 210 (4.66%) having the highest and the only percentage missing data in adolescent HIV testing data set. These missing values were imputed using the simple imputation technique. To prepare the data for the model, it was necessary to convert input and output features into numerical values. This was accomplished by utilizing the one-hot encoder to encode categorical variables found in the dataset. The dataset consisted of thirty one features with a single target feature. One-hot encoding is a valuable encoding technique for classification tasks. This technique transformed each categorical value into a new column with one-hot encoding, and the label values were created as new columns with values of either 1 or 0.

### Imbalance data handling

The current study aimed to address the issue of data imbalance and enhance the effectiveness of machine learning algorithms. Various techniques were employed to balance the dataset. The outcome feature revealed that the majority of observations (65.5%) were classified as not tested for HIV, while a smaller portion (34.5%) were tested. To balance the dataset, under-resampling, SMOTE, and ADASYN techniques were utilized, each employing a different approach to either maximize the minority class or decrease the majority class. The performance of each balancing technique was compared using selected supervised classification machine learning algorithms, with a focus on accuracy and AUC. In the unbalanced dataset, the random forest algorithm achieved a higher AUC of 84.9% compared to other classifiers, while the J48 decision tree had a higher accuracy of 82.4%. In the ADASYN approach, the J48 decision tree classifier achieved a higher accuracy of 83.1%, while logistic regression achieved a higher AUC of 79.9%. When comparing different balanced sampling methods using a J48 decision tree classifier, the SMOTE sampling technique performed the best, with an accuracy of 89.3% and an AUC of 86.3%. ADASYN was the second most effective method, with an accuracy and AUC of 83.1 and 76.9%, respectively. However, the under-sampling technique was found to be the least effective, with an AUC of 73.4% and an accuracy of 78.2% (Table 4).

**Table 4:** Compares imbalanced data handling techniques using accuracy and area under the curve.

Algorithms	Comparison method	Unbalanced	SMOTE	Under-sampling	ADASYN
Random forest	Accuracy	69.7	72.3	81.0	75.4
	AUC	71.9	77.9	69.6	70.6
Logit Boost	Accuracy	78.2	74.8	82.6	75.8
	AUC	74.0	81.0	69.8	72.7
J48 decision tree	Accuracy	82.4	89.3	78.2	83.1
	AUC	79.6	86.3	73.4	76.9
Naïve Bayes	Accuracy	65.7	73.9	69.7	82.5
	AUC	69.2	81.3	75.3	71.0
SVM	Accuracy	67.0	72.7	76.4	65.4
	AUC	70.8	66.8	73.5	62.5
KNN	Accuracy	70.5	68.7	69.0	70.3
	AUC	69.8	67.8	70.3	67.3
LR	Accuracy	75.0	75.2	78.8	69.9
	AUC	702	82.1	65.3	73.0
MLP	Accuracy	63.7	71.4	75.6	70.8
	AUC	81.0	76.4	73.4	67.5

**Figure 3:**
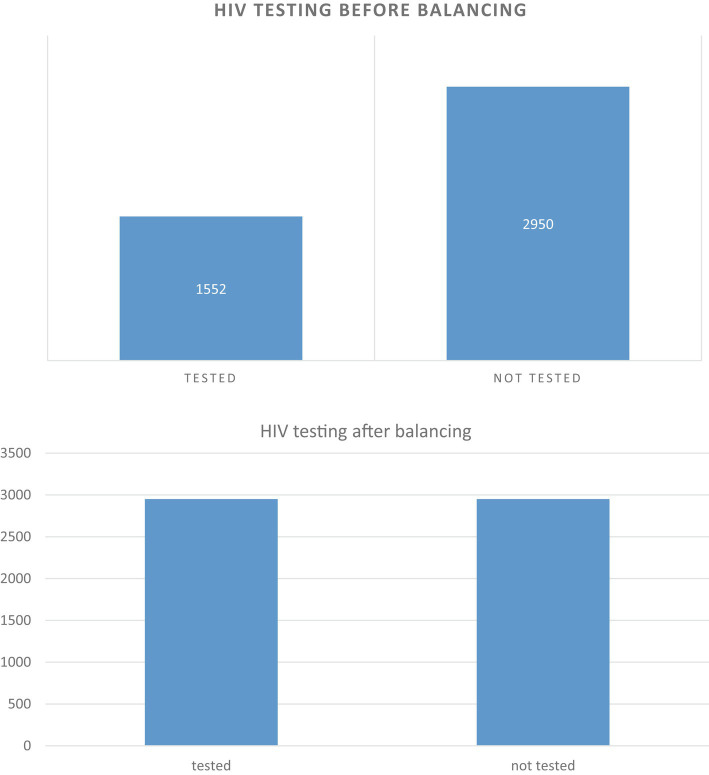
Before unbalanced and after balancing data of the target features.

Overall, the study demonstrated that employing balanced sampling methods can significantly improve the performance of machine learning algorithms on imbalanced datasets. The results suggest that SMOTE and ADASYN are effective techniques for balancing imbalanced data, while under-sampling may not be the most effective approach. The study in question faced a significant issue of imbalanced data, which could potentially hinder the performance of the classifying algorithm. To tackle this problem, a balanced sampling method was deemed crucial. Unbalanced data poses a challenge to machine learning, as values from the minority class or rarely occurring classes may be mistakenly classified as instances of the majority class. To address this issue, the study applied SMOTE to the unbalanced dataset, resulting in an increase in the total number of records (Figure 3). The classifier and the balanced sampling method were compared with other classifiers using training accuracy and AUC. Therefore, the study primarily relied on AUC to compare the classifier and the balanced sampling method. Overall, the use of a balanced sampling method proved to be effective in overcoming the issue of imbalanced data, and the study’s findings highlight the importance of considering AUC when evaluating the performance of classification algorithms on imbalanced datasets.

### J48 decision tree model performance

In this experiment, the objective was to evaluate the effectiveness of different classifiers in predicting the HIV testing status of adolescents. The primary goal of the analysis was to assess the accuracy of the predictions made by the chosen classifier. Among the classifiers that were selected, the random forest classifier exhibited particularly robust performance on the balanced dataset compared to the others. Once the best model was identified, hyper-parameter tuning and feature selection were performed. Additionally, the significance of the predictor for viral failure was determined to further evaluate the model’s performance.

### J48 decision tree with selected features

**Figure 4:**
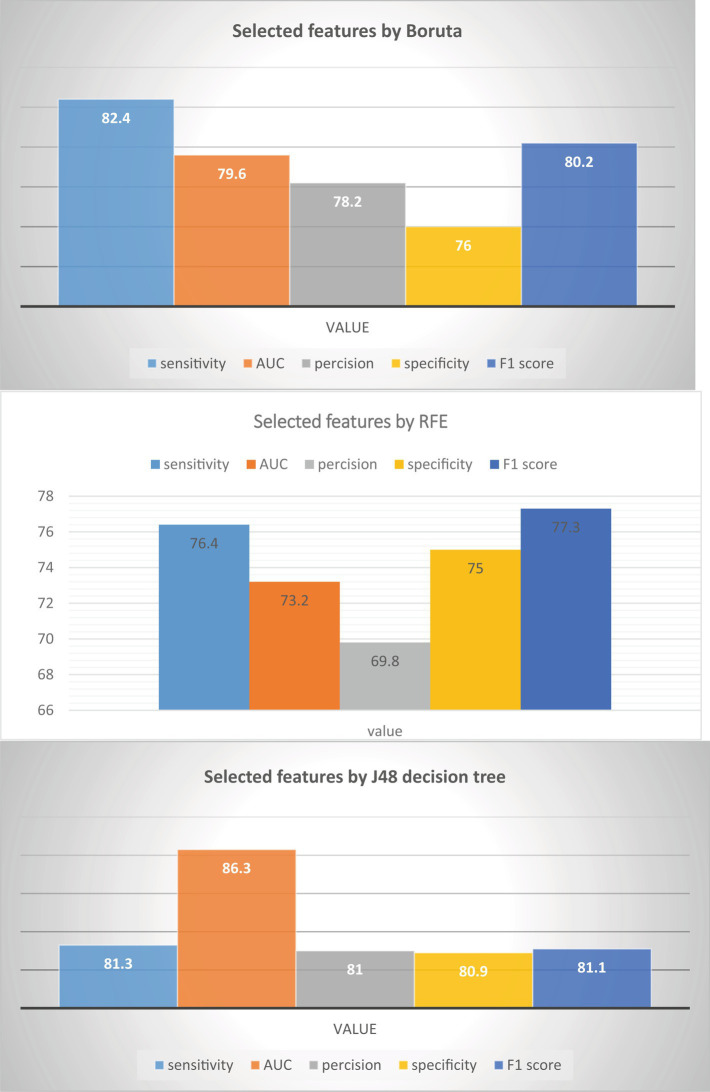
Comparison features selection method in adolescent HIV testing in Ethiopia.

This study aimed to assess the effectiveness of the J48 decision tree classifier in predicting HIV testing among adolescents and compare it with two other feature selection methods: Boruta feature selection and recursive feature elimination. The findings, as depicted in Figure 4, revealed that the J48 decision tree feature importance method outperformed both Boruta feature selection and recursive feature elimination. It achieved impressive results, including a sensitivity of 81.3%, specificity of 80.9%, precision of 81.0%, f1-score of 81.14%, and an AUC of 0.863. These outcomes strongly suggest that the J48 decision tree feature importance method is the most suitable approach for accurately predicting adolescent HIV testing. Conversely, Boruta feature selection and recursive feature elimination were found to be less effective in this context. Consequently, the J48 decision tree classifier was selected as the feature selection method for this study. Among all the features examined, the most significant predictors of HIV testing were age, knowledge of the place for HIV testing, age at first sexual encounter, recent sexual activity, exposure to family planning, and the number of sexual partners (see Table 5).

**Table 5:** Features degree of importance in predicting HIV testing status of the adolescents in Ethiopia.

S/N	Features name	Feature value	S/N	Features name	Feature value
1	Age	0.31733	11	Awareness on STI	0.15434
2	Know place of HIV testing	0.31543	12	Knowledge on contraceptive	0.14893
3	Age at first sex	0.28301	13	Knowledge on ovulatory cycle	0.14607
4	Recent sexual activity	0.26688	14	Literacy	0.13507
5	Exposure to family planning	0.24512	15	Wealth index	0.1285
6	Number of sexual partner	0.2015	16	Marital status	0.12292
7	Media exposure	0.1997	17	Alcohol drinking	0.11765
8	Residence	0.1916	18	Intimate partner violence	0.11203
9	Educational level	0.18108	19	Household members	0.08716
10	Awareness on AIDS	0.16044	20	Number of children	0.08367

### J48 decision tree with hyperparameter tuning

**Figure 5:**
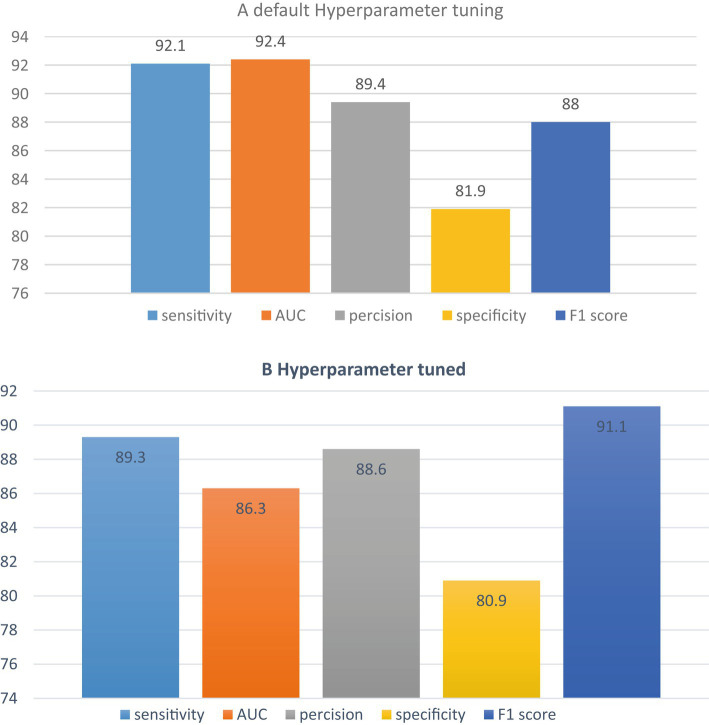
Comparison of tuned and default hyper parameter classifier.

After carefully selecting the most suitable classifier, this study proceeded to apply hyperparameter tuning or optimization to compare it with the default hyperparameter settings. Figure 5 visually represents the performance of the default hyperparameter configuration against the hyperparameter tuning approach when using the random forest classifier. The results indicated that the default hyperparameter configuration performed better than the tuned hyperparameters. Based on these findings, it was concluded that the random forest classifier with default hyperparameters outperformed the one with tuned hyperparameters. Consequently, the study decided to utilize the random forest classifier with default hyperparameter settings. The J48 decision tree classifier, after undergoing hyperparameter tuning, achieved impressive metrics, including a precision of 88.6%, an f1-score of 91.1%, a sensitivity of 89.3%, and a specificity of 80.9%. It is worth noting that the J48 decision tree classifier with hyperparameter tuning outperformed all other classifiers in terms of the area under the curve (AUC). In summary, the study ultimately presented the default hyperparameter tuning and hyperparameter tuned shown in Figure 5.

### Feature selection methods

In our analysis of the dataset, we utilized the Pearson Correlation Coefficient (PCC) as another feature selection method. PCC is a statistical measure that helps determine the correlation between two random attributes. The correlation value, represented by “r,” ranges from −1 to +1. By applying PCC to measure the correlation between the attributes and the target variable, which in this case is “adolescent HIV testing,” we were able to identify which features were positively or negatively correlated with the target class. The results of this analysis are presented in Table 6, which lists the correlation values of each feature with respect to the target variable. The feature with the highest correlation value is “know the place of HIV testing,” with a value of 0.337. Additionally, “age” exhibits a correlation of 0.317, while “age at first sex” has a correlation of 0.306 (Table 6). A positive correlation indicates that the variables are positively associated, meaning that as the value of x increases, the value of y also increases, and vice versa.

**Table 6:** The correlation of predictors to adolescent HIV testing attributes.

Feature	Correlation value	Feature	Correlation value
Residence	−0.192	Age	0.317
Educational level	0.294	Total children ever born	0.084
Region	0.130	Number of household member	0.165
Sex of household head	−0.008	Number of living children	0.080
Literacy	0.171	Recent sexual activity	0.271
Wealth index	0.236	Currently working	0.059
Knowledge of ovulatory cycle	0.146	Awareness on STI	0.154
Knowledge of any contraceptive method	0.149	Worked in last 12 months	0.062
Respondent circumcised	0.013	Ever heard of AIDS	0.160
Current marital status	0.123	Alcohol drinking	0.117
Age at first sex	0.306	Intimate partner violence	−0.112
Know a place to get HIV test	0.337	Media exposure	0.200
Have you ever chewed Chat?	0.057	Stigma and discrimination	0.050
Exposure to family planning	0.245	Number of Sexual partner	0.198
Smoking status	0.032	Health insurance	0.026
Resource control	0.071		

After studying the correlation of the predictors to the target, we also took into consideration the collinearity. Collinearity happens when two predictors are linearly associated or having a high correlation to each other, and both were used as predictors of the target variable (21). Multicollinearity may also happen, which is a situation wherein the variable has collinearity with more than one predictors in the dataset. We used the Variance Inflation Factor (VIF) to detect the collinearity of the predictors in the dataset. The VIF starts from 1 to infinity, and the value of 1 means that the features were not correlated. VIF values less than 5 are moderately correlated, while VIF values of 10 and above are highly correlated and a cause of concern (21). The VIF values of each predictor in the dataset can be seen in Table 7.

**Table 7:** VIF of predictors in the dataset.

Feature	VIF value	Feature	VIF value
Residence	2.063	Age	1.442
Educational level	1.383	Total children ever born	25.109
Region	1.312	Number of household member	1.108
Sex of household head	1.024	Number of living children	1.301
Literacy	1.460	Recent sexual activity	3.624
Wealth index	1.415	Currently working	1.192
Knowledge of ovulatory cycle	1.057	Awareness on STI	1.215
Knowledge of any contraceptive method	1.097	Worked in last 12 months	16.599
Respondent circumcised	1.015	Ever heard of AIDS	1.213
Current marital status	3.294	Alcohol drinking	1.145
Age at first sex	1.375	Intimate partner violence	1.088
Know a place to get HIV test	1.109	Media exposure	1.372
Have you ever chewed Chat?	1.144	Stigma and discrimination	1.034
Exposure to family planning	1.214	Number of Sexual partner	1.596
Smoking status	1.014	Health insurance	1.062
Resource control	1.378		

**Figure 6:**
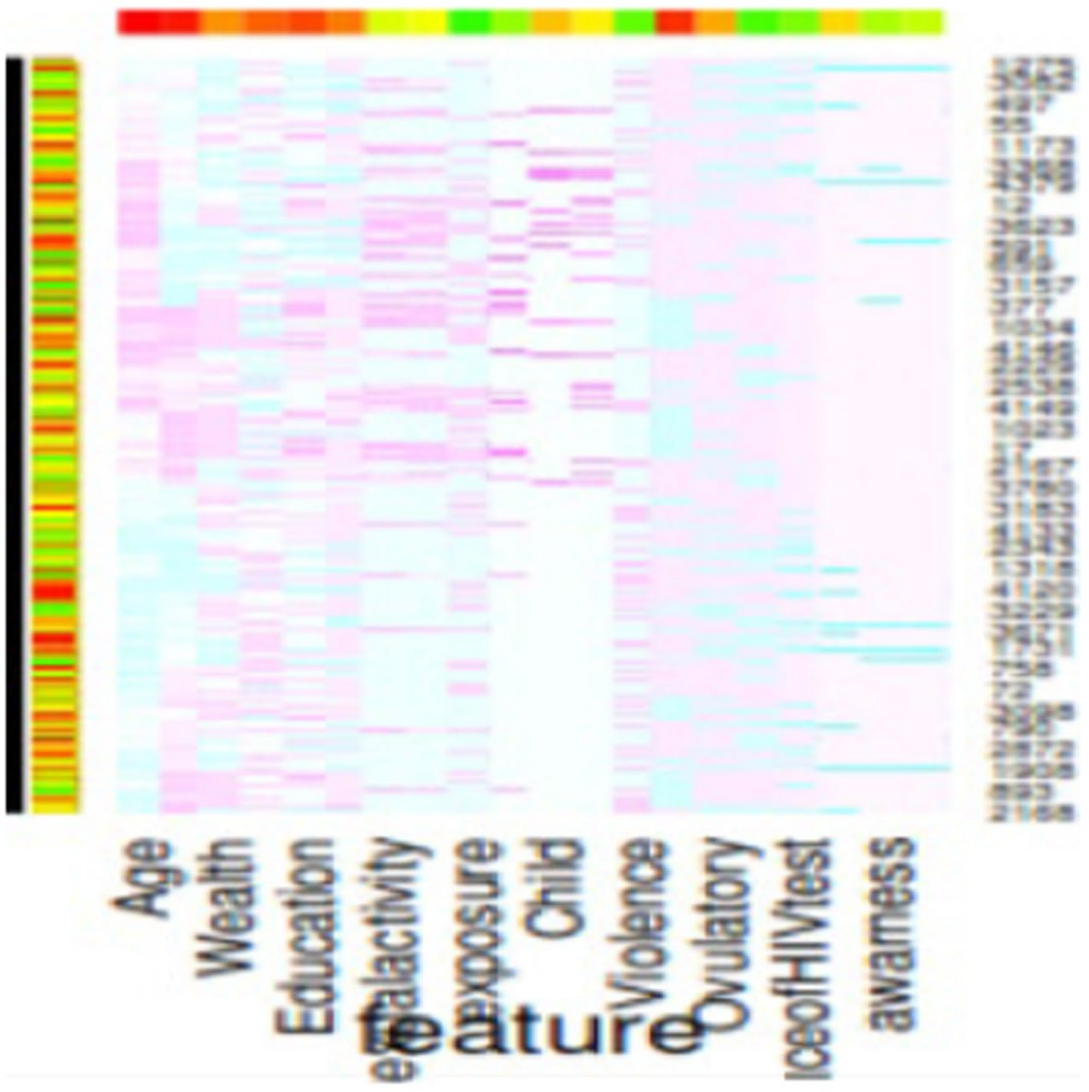
SHAP plot of features selected.

Table 7 presents the Variance Inflation Factor (VIF) for each predictor in the dataset. The highest VIF value is observed for the variable “total children ever born,” which stands at 25.109. Other predictors such as “working in the last 12 months,” “current marital status,” “recent sexual activity,” and “residence” also exhibit relatively high VIF scores, although they are lower than 5. A VIF between 1 and 5 suggests that these predictors are not strongly correlated and can be considered for inclusion in the adolescent HIV testing model. Therefore, it is recommended to include “working in the last 12 months,” “current marital status,” “recent sexual activity,” and “residence” as predictors when building the adolescent HIV testing model. In addition, we used SHAP plot of feature selection. Based on SHAP plot age, wealth, sexual activity, educational status, exposure to family planning, IPV, know the place of HIV testing and awareness on STI and AIDS were identified (Figure 6).

### Data analysis and feature selection

A comprehensive literature review has thoroughly examined 31 features that contribute to HIV testing services for adolescents. Through a feature evaluator, the significance of these factors was assessed, resulting in the identification of 20 highly important variables during the feature selection process. However, only 20 features were included in the analysis, while others were excluded based on specific criteria outlined in Figure 1.

To predict the HIV testing status of adolescents, the significance of each factor was calculated, leading to the selection of 20 predictors for machine learning (ML) algorithms. Among these predictors, age emerged as the most important factor for HIV testing services, with a value of 0.31733. On the other hand, the total number of children born was found to be the least important predictor, with a value of 0.08367. The importance of each feature in the dataset was calculated and presented in Table 5, displaying the variables in descending order of ranking.

### Developing and evaluating models

**Figure 7:**
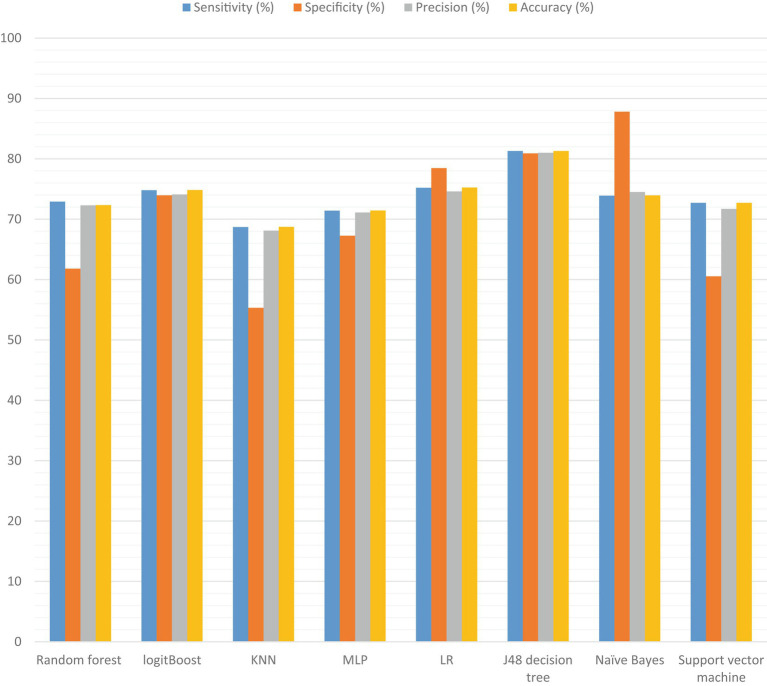
Visual comparisons of ML algorithm capabilities for adolescent HIV testing services.

**Figure 8:**
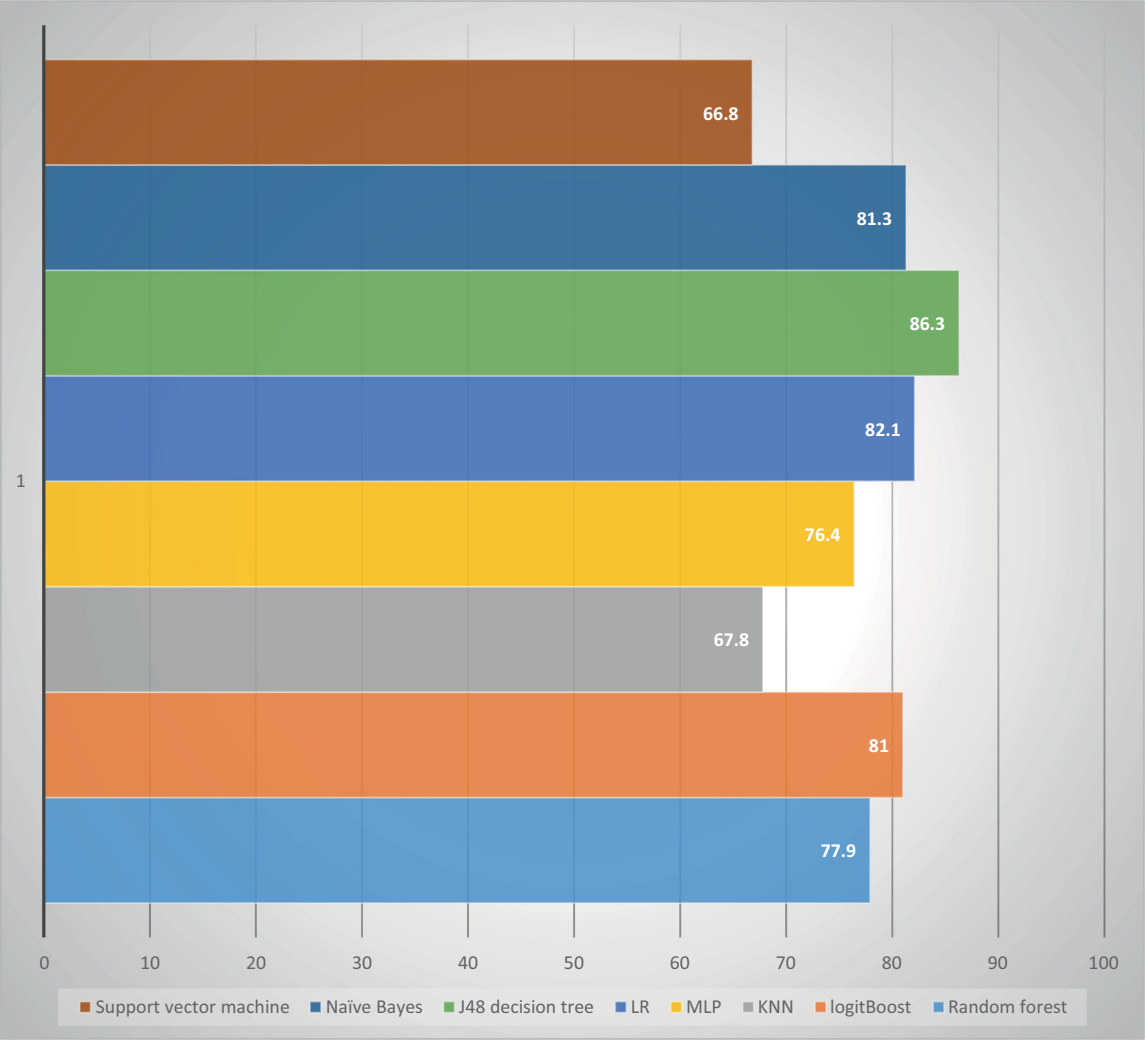
Comparison of ROC area under the curve in the study conducted among adolescents HIV testing status in Ethiopia.

**Figure 9:**
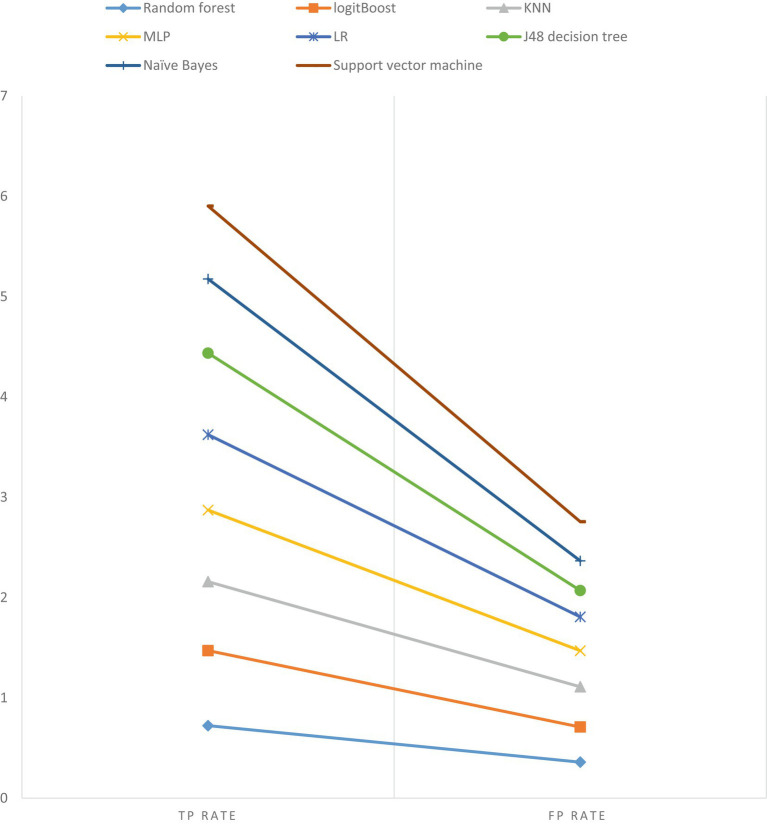
The true positive rate and false positive rate of the eight algorithms.

In this study, our objective was to predict HIV testing among adolescents by selecting the most optimal features and utilizing eight different machine learning (ML) algorithms. These algorithms included J48, RF, LR, MLP, logit Boost, k-NN, SVM, and NB. To evaluate the performance of each algorithm, we conducted 10-fold cross-validation with a seed value of two and assessed various metrics, such as sensitivity, specificity, accuracy, precision, and the receiver operating characteristic (ROC) curve. The results of the cross-validation are presented in Table 8. Our experimental findings revealed that the J48 decision tree algorithm outperformed the other ML algorithms in accurately predicting adolescent HIV testing. It achieved impressive performance metrics, including a sensitivity of 81.30%, specificity of 80.90%, accuracy of 81.3%, precision of 81.0%, and an ROC value of 86.3%. Figure 7 visually depicts the performance metrics of the ML algorithms used in this study, while Figure 8 presents a comparison of the area under the ROC curve for these algorithms. Notably, the SVM algorithm exhibited the lowest performance with an ROC value of 66.8% according to the ROC analysis. Figure 9 presents the false positive rate and true positive rate of each algorithm. For a comprehensive summary of the performance evaluation of each algorithm, please refer to Table 8.

**Table 8:** Performance evaluation of the selected ML algorithms for HIV testing prediction.

Algorithm	Sensitivity (%)	Specificity (%)	Precision (%)	Accuracy (%)	Area under ROC (%)	F1 score
Random forest	72.90	61.8	72.3	72.3234	77.9	72.598
Logit boost	74.80	73.96	74.10	74.8334	81.0	74.448
KNN	68.70	55.32	68.1	68.725	67.80	68.398
MLP	71.4	67.26	71.1	71.4349	76.4	71.249
LR	75.2	78.47	74.6	75.2332	82.1	74.898
J48 decision tree	81.3	80.9	81.0	81.2972	86.3	81.149
Naïve Bayes	73.9	87.80	74.5	73.9449	81.3	74.198
SVM	72.7	60.53	71.7	72.701	66.8	72.196

### Association rule result

This study utilized the J48 decision tree feature importance method to select relevant features. Subsequently, the association mining rules were applied using the apriori algorithm for interpretation and a comparison of the best-selected features. From the association mining rules, a total of nine rules were identified with a confidence value of over 90% and the highest lift or interestingness. Among these rules, the twenty most significant ones were chosen for predicting adolescent HIV testing. The absolute minimum support count of the apriori algorithm was 2,251 in support of 0.5 and confidence of 0.9. The summary of quality measures of association rule mining analysis indicated that minimum support value = 0.5018, confidence = 0.9461, lift = 0.9924. The total rules of this apriori algorithm of this study was 712,602 done by 48 s.

Rule 1 indicated that individuals know the place of HIV testing (yes), intimate partner violence (=no), had media exposure (=yes), number of sexual partner (=more than one), awareness on AIDS (=yes), awareness on STI (=yes), sexual activity (=active in last 4 weeks), age at first sex (=between16 to 24 years), marital status (=married), knowledge on contraceptive (=knowledgeable), number of children (=one and above), knowledge on ovulatory cycle (=yes) and exposure to family planning (=yes) then the possibility of HIV testing was 95.33% of confidence with support value = 95% and Lift value = 31.444.

Rule 2 means that being rural residence, awareness on AIDS (=yes), awareness on STI (=yes), sexual activity (=active in last 4 weeks), age at first sex (=between16 to 24 years), marital status (=married), knowledge on contraceptive (=knowledgeable), number of children (=one and above), knowledge on ovulatory cycle (=yes) and literacy (able to read and write) the adolescent will be 92.24% chance of HIV testing with support = 93% and Lift = 33.334.

Rule 3 means that if age greater than 18 years old, education (=secondary), age at first sex (16–24 years), recent sexual activity (active in last 4 weeks), exposure to family planning (=yes), and had media exposure the probability of adolescent HIV testing is 100% of confidence with support of 0.00255, Lift = 31.233%.

Rule 4 showed that if the adolescent attended secondary education, age above 18 years, intimate partner violence (=no), had media exposure, number of sexual partner (more than one), had awareness on AIDS, and had awareness on STI a then the possibility of the adolescent for testing HIV is 100% of confidence, support = 0.00222, Lift = 33.377.

Rule 5 means that if the adolescent attended secondary education, know the place of HIV testing, age at first sex (16–24), urban residence, and had knowledge on contraceptives then the possibility of adolescent HIV testing is 100% confidence and support = 0.001033, Lift = 32.64.

According to the association rule analysis, it was found that several factors strongly predict adolescent HIV testing in Ethiopia. These factors include knowing the place of HIV testing, being above 18 years old, having a secondary education or higher, engaging in first sexual activity above the age of 16, living in urban areas, having recent sexual activity, being exposed to family planning information, having more than one sexual partner, being aware of STIs and AIDS, and having media exposure. The association rules indicate a strong relationship between these independent features and the dependent feature (adolescent HIV testing). This is supported by the lift values, which are greater than one. This suggests that the presence of these factors increases the likelihood of adolescents getting tested for HIV in Ethiopia. In summary, the association rule analysis highlights the significant predictors of adolescent HIV testing in Ethiopia, emphasizing the interconnections between various factors and the importance of these factors in promoting HIV testing among adolescents.

## Discussion

In this study, the researchers aimed to develop a highly accurate machine-learning model for predicting HIV testing among adolescents in Ethiopia. What sets this research apart is its focus on exploring new predictor variables that have not been previously investigated. To achieve this, the researchers analyzed secondary data from the demographic and health survey of Ethiopia, which provided them with relevant information on the testing status of adolescents, their medical history, and demographic characteristics.

The researchers conducted a comprehensive study on predicting HIV testing status using various statistical analysis techniques and feature selection methods. They utilized a range of machine-learning models, including J48 decision tree, RF, k-NN, MLP, NB, logit Boost, SVM, and LR models. Among these techniques, the J48 decision tree model demonstrated the highest performance, achieving an accuracy of 81.29%. It also showed a sensitivity of 81.30%, precision of 81.0%, specificity of 80.90%, and an ROC of approximately 86.30%. These results indicate that the J48 decision tree is an exceptionally effective machine-learning technique for this specific task. The study also revealed that the J48 decision tree, KNN, MLP, LR, Naïve Bayes, and XGBoost models exhibited good prediction performance, with ROC values above 76.4%. These models also demonstrated superior diagnostic efficiency compared to other models trained with the same parameters. Overall, this research provides valuable insights into the development of machine-learning models for predicting HIV testing services among adolescents. Implementing these models could potentially enhance adolescent HIV testing status and reduce the number of undiagnosed chronic HIV carriers in the country.

Age has the most significant impact on an individual’s HIV testing status compared to other factors. Any change in age can have a more pronounced influence than other variables. Older individuals are more likely to undergo HIV testing. Moreover, individuals who are aware of the locations where HIV testing is available have a higher likelihood of getting tested. Additionally, the age at which adolescents first engage in sexual activity also plays a role in their HIV testing behavior in Ethiopia. This could be due to increased access to information and improved decision-making abilities among adolescents. Furthermore, it appears that those who are sexually active are more likely to seek HIV testing compared to their counterparts. Individuals with lower levels of education tend to have less knowledge about HIV risk mitigation measures, thereby increasing their vulnerability to HIV. This highlights the importance of HIV testing services in providing individuals with knowledge and awareness about HIV prevention.

Recent studies have also investigated the potential predictive algorithms for HIV testing services among adolescents. For instance, Mutai et al. (43) developed a prediction model using six machine-learning techniques, with the XGBoost algorithm showing promising results. They identified factors such as sex, age, relationship with family head, highest level of education, highest grade at school level, work for payment, avoiding pregnancy, age at first experience of sex, and wealth quintile as having the highest weights in their model. These findings align with our own research, which predicts HIV testing status among adolescents based on nationally representative data. Another study involving 6,346 men who have sex with men indicated that the RF algorithm performed best, with an AUC of 0.942 compared to other three machine learning algorithms (50). Overall, these studies contribute valuable knowledge to the field of HIV testing prediction models, offering insights into the potential factors and machine-learning techniques that can be utilized to improve testing services among adolescents.

Several research studies have investigated the application of machine learning (ML) techniques for predicting HIV testing and HIV infection among different population category in different countries. A longitudinal study conducted among 2,564 adolescent indicated that Random forest was the best model of predicting risky behaviour of the adolescents with AUC = 0.84 on training data and 0.87 on testing data as compared with other four algorithm of the study (51). Based on other study conducted on HIV and STI testing at a clinic, ten algorithms were used for prediction testing (52). Out of those ten algorithms, the XGBoost model demonstrated the highest accuracy with an AUC of 62.8% and an F1 score of 70.8%. In another study that involved 55,151 males and 69,626 females in East and South Africa, the gradient boosting trees algorithm was found to be the most effective in predicting HIV status (53). This implies that machine-learning was the best predictor of testing services and identifies the best features of the outcome.

A 81.3% sensitivity was required in ensuring that 81.3% of individuals tested and knew their status. With the J48 decision algorithm, we utilized 20 most predictive variables accordingly to establish the number required to screen to know one individual with HIV. There community-based and facility-based screening were studies in previous literatures (54). This implies J48 decision tree predicts the testing of adolescent in Ethiopia. Our study revealed that the J48 decision tree model algorithm outperformed other models, emerging as the top performer. Interestingly, our findings differ from Orel’s research in terms of the predictors identified, except for the consistent identification of individual age and wealth as predictors of the disease (55). The possible justification for variation could be variation in demographic characteristics of the study participants.

There are alternative screening methods available; however, they do come with certain limitations. Universal screening, for instance, involves conducting tests on all patients in healthcare facilities. While this approach can be effective, it may not be cost-effective in situations where the incidence of the condition being screened for is low like Ethiopia (56). Indicator-condition-guided testing, which fails to consider important factors such as age, sex, and medical conditions, overlooks their association with a reduced risk of HIV transmission (57). In settings where HIV prevalence is high, it is effective to target well-established risk groups, such as families (through index contact elicitation), to reach individuals at high risk (58). However, this approach may unintentionally neglect lesser-known or harder-to-define subgroups that are also vulnerable (59), resulting in inefficient resource allocation (60). Even in the absence of recognized risk factors, self-assessment provides a means of identifying individuals at high risk. Therefore, it is crucial to consider targeted testing and demographic factors like age in order to achieve the first 95% of the UNAIDs target in the country.

An individual’s perception of risk is influenced by their level of awareness about HIV and its related factors (61). Sometimes, unexpected or uncontrolled exposures to the virus can go unnoticed. In the case of a widespread outbreak, it may not be obvious which demographic subgroups should be targeted for prevention efforts. Merely providing Pre-Exposure Prophylaxis to established high-risk subgroups, such as young people or mobile populations, may not be effective. Therefore, it is important to consider a PrEP technique that takes into account individual characteristics in a more nuanced way (62). This approach could help reduce the cost of preventing new HIV infections. Our method offers an alternative to the limitations mentioned earlier and could potentially complement existing strategies for identifying individuals who would benefit the most from enhanced mitigation measures.

## Conclusion

In our study, we utilized country-level demographic and health services data to develop a groundbreaking model for predicting adolescent HIV testing services. This model incorporates demographic characteristics that were carefully developed after an extensive review of a large dataset, showcasing its superior predictive capacity compared to existing literature. The main objective of our model is to prioritize early identification of barriers to testing high-risk patients and optimize the utilization of strained public healthcare systems.

We strongly believe that our proposed technique has the potential to significantly enhance healthcare systems’ decision-making processes, enabling precise and targeted HIV testing strategies in countries. This, in turn, empowers adolescents by promoting HIV testing and designing well-organized testing strategies. Our study specifically focused on creating and evaluating machine learning-based prediction models for HIV testing services in Ethiopia, utilizing 20 key predictors. Among the eight machine learning algorithms tested, the J48 decision tree model demonstrated the highest classification accuracy and precision. This suggests that our proposed model can effectively predict adolescent HIV testing, thereby optimizing the allocation of limited resources in the country and surpassing the achievement of the 95–95-95 target. Importantly, our model identified age as the highest predictor of adolescent HIV testing, while the number of children ever born was the lowest predictor.

In conclusion, the integration of machine learning algorithms with comprehensive national-based data enables accurate classification of individuals for HIV testing services. This advancement holds great promise in improving healthcare outcomes and resource management, particularly during the ongoing pandemic. Surveys providing detailed individual-level data, including demographic characteristics, social history, laboratory tests, and disease results, have become more available. Leveraging these datasets through advanced approaches can significantly aid in the prevention, diagnosis, and testing of HIV and other diseases. By incorporating this approach into community-based or facility-based testing programs, it becomes possible to identify individuals at high risk. However, further studies are required to refine this model, effectively integrate it, and apply it in real-world primary care settings.

### Limitation and strength

This retrospective study analyzed demographic and health survey data that exhibited irregularities and imbalances. To address this issue, the researchers took measures to balance the dataset by removing noisy and inadequate records. Specifically, they focused on tackling the problem of imbalanced classes, where the number of records related to the HIV-tested class was significantly lower than the tested or never tested (1,552 vs. 2,950).

To evaluate the performance of each machine learning algorithm, various criteria were utilized. Furthermore, external validation of the proposed model was conducted using multi-center country-level data, aiming to enhance the generalizability of the predictions. However, it is important to note that the researchers relied on self-reported data from the demographic and health survey, which may introduce inconclusiveness and potentially impact the training data.

For future research, the researchers recommend that behavioral features be incorporated to further enhance prediction accuracy. Additionally, it is crucial to monitor the dynamic variations of significant features over time to better identify adolescents HIV testing in the countries. While the study provides valuable insights using the available data, there are areas for improvement and avenues for further research to enhance the understanding and prediction of HIV testing services in this specific sub-population group in Ethiopia.

## Data availability statement

The datasets presented in this study can be found in online repositories. The names of the repository/repositories and accession number(s) can be found in the article/Supplementary material.

## Ethics statement

Ethical approval was not required for the study involving humans in accordance with the local legislation and institutional requirements. Written informed consent to participate in this study was not required from the participants or the participants’ legal guardians/next of kin in accordance with the national legislation and the institutional requirements.

## Author contributions

MA: Conceptualization, Data curation, Formal analysis, Methodology, Resources, Software, Validation, Visualization, Writing – original draft, Writing – review & editing. YN: Data curation, Formal analysis, Investigation, Methodology, Validation, Visualization, Writing – original draft, Writing – review & editing.

## Footnotes

^1^https://dhsprogram.com/
