# Supplementary figures and images for: Machine learning prediction of adolescent HIV testing services in Ethiopia
Source: Front Public Health. 2024 Mar 15;12:1341279. doi: 10.3389/fpubh.2024.1341279 (PMC10981275; doi:10.3389/fpubh.2024.1341279)

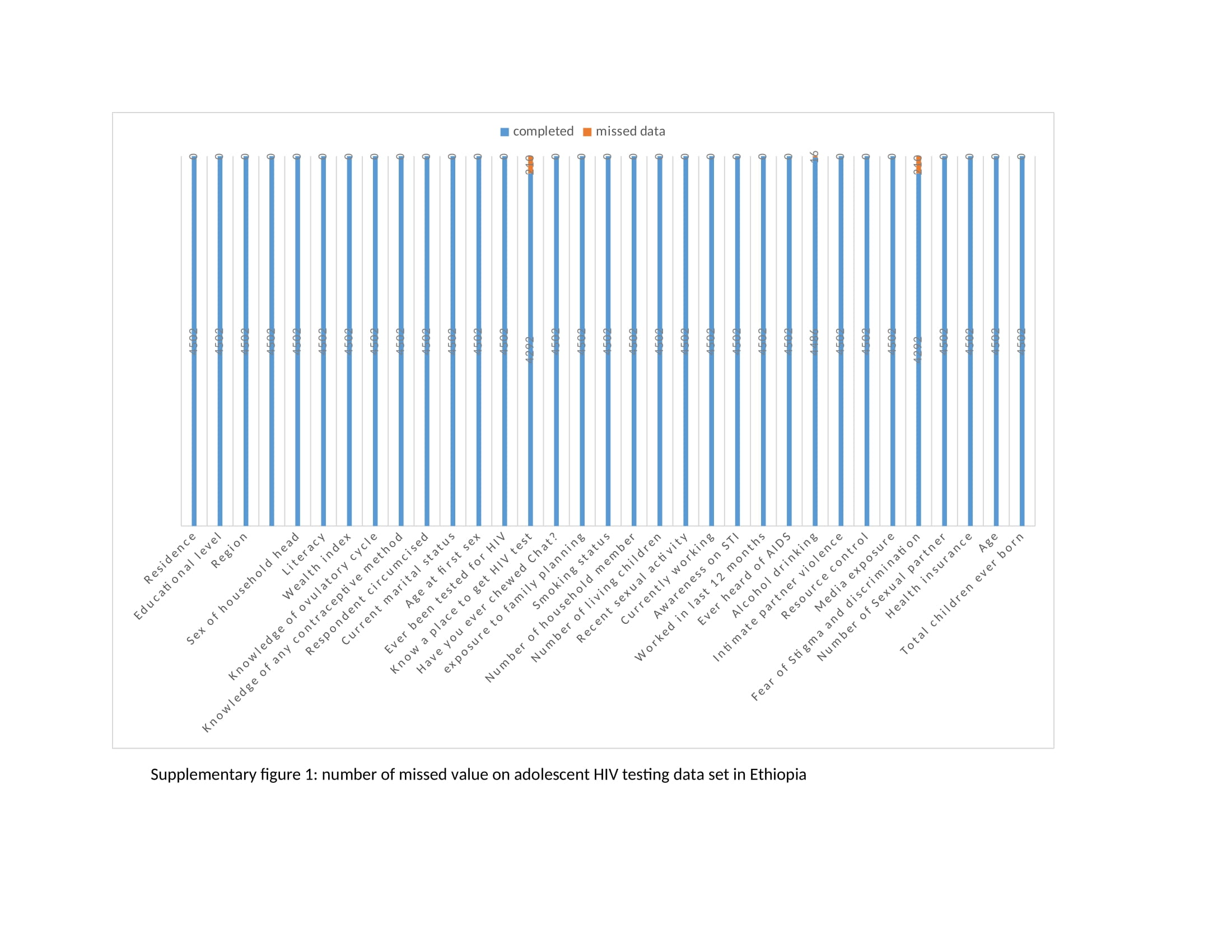

Supplement: Supplementary file 1 [file Image_1.jpg]
